# Physical Activity as a Tool for Social Inclusion in Multiple Sclerosis: A Systematic Review of Qualitative, Quantitative, and Mixed-Methods Evidence

**DOI:** 10.3390/sports14010025

**Published:** 2026-01-05

**Authors:** Federica Marzoli, Ludovica Cardinali, Gianluca Di Pinto, Matteo Campanella, Andrea Colombo, Dafne Ferrari, Lorenzo Marcelli, Fioretta Silvestri, Andrea De Giorgio, Andrea Velardi, Davide Curzi, Laura Guidetti

**Affiliations:** 1 Department of Economic, Psycological, Communication, Education and Movement Sciences, University “Niccolò Cusano”, 00166 Roma, Italylaura.guidetti@unicusano.it (L.G.); 2 Department of Life Science, Health, and Health Professions, Link Campus University, 00165 Roma, Italyd.ferrari@unilink.it (D.F.); 3 Department of Medicine and Aging Sciences, University “G. D’Annunzio” Chieti-Pescara, 66100 Chieti, Italy; 4 Department of Theoretical and Applied Sciences, eCampus University, 22060 Novedrate, Italy; 5 Department of Political, Legal, Sociological, and Human Sciences, University “Niccolò Cusano”, 00166 Roma, Italy

**Keywords:** multiple sclerosis, physical activity, exercise, social barriers

## Abstract

**Background**: People with multiple sclerosis (PwMS) face a wide range of social barriers, including stigma, limited support, and inaccessible environments, that restrict participation in physical activity (PA). Although PA is known to improve physical and psychological outcomes, its role in reducing social barriers has not been clearly synthesized. **Methods**: Following PRISMA 2020 guidelines, we conducted a systematic search of PubMed, Scopus, and Web of Science (January 1997–October 2025). Qualitative, quantitative, and mixed-methods studies examining how PA relates to social barriers, facilitators, or social outcomes for PwMS were included. Data were synthesized using a thematic analysis approach due to heterogeneity in study designs and outcomes. Risk of bias was assessed using JBI, NIH, RoB 2.0, and MMAT tools. **Results**: Twenty-nine studies met the inclusion criteria. The thematic synthesis identified three overarching mechanisms through which PA contributes to reducing social barriers: (1) peer support and shared experience, whereby group-based PA reduced isolation and normalized fluctuating symptoms; (2) guidance from knowledgeable professionals, which fostered trust, confidence, and perceived safety; and (3) changes in social identity, with participants shifting from a “sick role” toward identities such as “exerciser” or “athlete.” These mechanisms were supported by high-quality qualitative studies and by quantitative evidence showing small-to-moderate effect sizes for improvements in self-efficacy, social participation, and perceived social support. **Conclusions**: PA functions as a socially transformative practice for PwMS when delivered in group-based, supervised, and accessible formats. Programs designed to intentionally cultivate peer connection, professional support, and identity-building processes may be especially effective in overcoming social barriers and promoting social inclusion.

## 1. Introduction

Multiple sclerosis (MS) is a chronic, immune-mediated neurological disorder and one of the leading causes of non-traumatic disability among young adults, with approximately 2.8 million individuals affected worldwide [1]. Its clinical presentation—including fatigue, motor impairment, cognitive difficulties and sensory disturbances–contributes to progressive functional decline and significantly reduces health-related quality of life (HRQoL) [2]. The unpredictable nature of MS poses challenges not only to physical functioning but also to psychological well-being and social participation [3,4]. Physical activity (PA) and structured exercise are recognized as essential non-pharmacological strategies for PwMS, with robust evidence documenting improvements in muscular strength, cardiovascular fitness, mobility, fatigue management and mood [3,4].

Psychological well-being (PWB) benefits include reductions in depressive symptoms, improved self-acceptance, and enhanced autonomy [5,6]. As a result, several PA guidelines tailored specifically to PwMS have been developed and widely disseminated [7].

Despite these benefits, PwMS remain significantly less active than the general population [8,9]. Meta-analysis evidence confirms that PwMS engage in markedly lower levels of PA compared with healthy controls [10], revealing an enduring gap between clinical recommendations and real-world participation. Although symptom severity, fatigue, and safety concerns are commonly cited as intrapersonal barriers [11], growing evidence demonstrates that social and environmental factors often exert an even stronger limiting influence [12,13,14].

A key distinction derived from disability studies differentiates impairment (i.e., the neurological symptoms of MS), and disability (i.e., the social and environmental restrictions that limit participation) [15]. Many barriers to PA fall into this second category: insufficient social support, stigmatizing attitudes, inaccessible facilities, transportation limitations, and the scarcity of professionals trained to work with MS [12,13]. These factors collectively produce a persistent “participation gap,” whereby PwMS are excluded from meaningful PA opportunities. These factors collectively produce a persistent ‘participation gap,’ whereby PwMS are excluded from meaningful PA opportunities [16].

While previous reviews have catalogued barriers and correlates of PA in MS, the field lacks a synthesis explicitly examining how PA itself functions as a mechanism to overcome social barriers. Theoretical frameworks such as Social Cognitive Theory (SCT) [17] and the Social–Ecological Model [18] offer lenses through which to interpret these dynamic interactions. SCT emphasized reciprocal influences between environment, cognitions (e.g., self-efficacy), and behavior, while socio-ecological models highlight structural barriers that constrain even the most motivated individuals.

Therefore, the aim of this systematic review is to synthesize the existing qualitative, quantitative, and mixed-methods literature to answer the following specific research question: *How does physical activity or exercise help people with multiple sclerosis overcome social barriers?* By identifying mechanisms that transcend study designs and intervention type, this review contributes to the development of interventions that are not only physically accessible but also socially transformative.

## 2. Materials and Methods

### 2.1. Study Design

This systematic review was conducted and reported in strict accordance with the PRISMA 2020 guidelines [19]. The completed PRISMA 2020 checklist is provided as Supplementary Materials. A structured and replicable protocol was followed to identify, select, and synthesize information on how physical activity (PA) may help people with multiple sclerosis (PwMS) overcome social barriers.

### 2.2. Eligibility Criteria

Study selection followed a pre-defined PICOS framework:

- Population: Adults (age ≥ 18) with a clinically confirmed diagnosis of Multiple Sclerosis (PwMS). Studies including mixed populations were eligible only when separate results for PwMS were reported.
- Intervention: Any form of PA, exercise, adapted exercise, recreational activities, or lifestyle PA. Studies were eligible if they examined social barriers (e.g., stigma, lack of support), social facilitators (e.g., peer support, group settings), or social consequences (e.g., identity change, social participation) related to PA.
- Comparator: Any comparator (e.g., control group, wait-list, baseline measurements, alternative intervention) or none, as appropriate for the study design.
- Outcomes: Eligible studies reported qualitative, quantitative, or mixed-methods data on social dimensions of PA. To improve reproducibility and conceptual clarity, social constructs were explicitly operationalized as follows:
  ○ Social support: perceived or received encouragement, companionship, or assistance (peers, family, healthcare professionals, community members).
  ○ Stigma: enacted stigma (discrimination, negative attitudes) and internalized stigma (self-identification with negative stereotypes such as “too sick to exercise”).
  ○ Social identity: perceptions of oneself in a social context, including shifts toward “exerciser”, “athlete” or “capable person”.
  ○ Social participation/inclusion: involvement in community activities, group programs, classes; perceived belonging and acceptance.
- Study Designs: eligible study designs included qualitative studies, quantitative observational studies (cross-sectional, cohort), randomized controlled trials (RCTs), non-randomized intervention studies, and mixed-methods studies.

Exclusion criteria were: (i) studies not published in English; (ii) studies not reporting original data (iii) studies focusing solely on physiological, biomechanical, or non-social psychological outcomes without a social context; (iv) conference abstracts, editorials, dissertations, gray literature; (v) studies on non-MS populations without separate data for PwMS. Two study protocols [20,21] were included because they provided conceptual and contextual frameworks relevant to social barriers and facilitators, despite not reporting empirical outcome data. Inclusion and exclusion criteria have been reported in Table 1.

### 2.3. Information Sources and Search Strategy

A comprehensive and systematic literature search was executed across three major electronic databases: PubMed, Scopus, and Web of Science. The final search was conducted on 31 October 2025, covering publications from January 1997. The complete search strategy for all databases is reported in Table S1 (Supplementary Materials). Preliminary scoping search indicated that publications addressing the social dimensions of PA in MS began appearing after 1997. Therefore, this starting point ensured coverage of all contemporary conceptualizations of social barriers. Only peer-reviewed research articles written in English, involving adults (≥18 years) human participants, were deemed eligible.

Full Search Strings:

PubMed:

("multiple sclerosis"[MeSH Terms] OR "multiple sclerosis"[Title/Abstract]) AND ("physical activity"[Title/Abstract] OR exercise [Title/Abstract] OR sport [Title/Abstract]) AND ("social barriers"[Title/Abstract] OR "social support"[Title/Abstract] OR stigma [Title/Abstract] OR "social participation"[Title/Abstract] OR identity [Title/Abstract])

Scopus:

TITLE-ABS-KEY (("multiple sclerosis") AND ("physical activity" OR exercise OR sport) AND ("social barriers" OR "social support" OR stigma OR "social participation" OR identity))

Web of Science:

TS = ("multiple sclerosis") AND TS= ("physical activity" OR exercise OR sport) AND TS = ("social barriers" OR "social support" OR stigma OR "social participation" OR identity)

The reference management software Zotero (version v 7.0.22, 2025) (Fairfax, VA, USA) was used to import, manage and analyze the retrieved articles.

### 2.4. Study Selection Process

**Figure 1:**
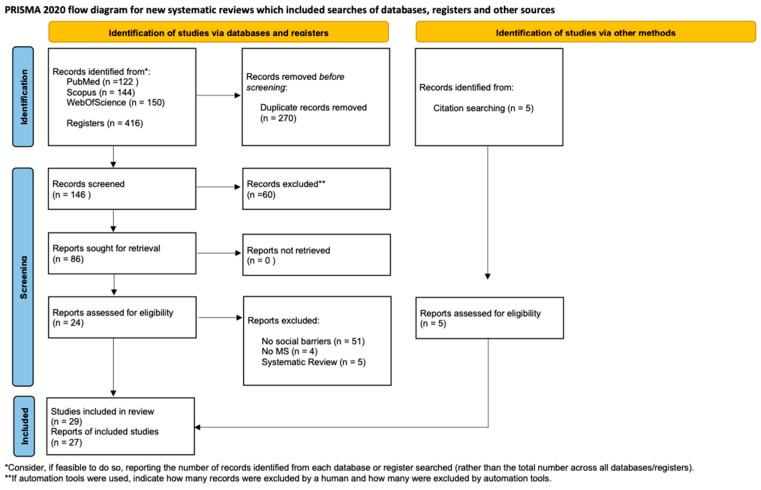
The PRISMA flow diagram [19].

Studies were included if they met the inclusion criteria. The study selection process followed the PRISMA 2020 flow diagram (Figure 1). All records identified through database searching were imported into the web-based systematic review tool Rayyan [22], where duplicates were removed using both automated and manual checks. Two independent reviewers (F.M., L.C.) conducted both Title and Abstract Screening and Full-Text Assessment. Disagreements were resolved by discussion and unresolved cases were adjudicated by a senior reviewer (L.G.). Inter-rater reliability for the full-text screening phase was calculated using Cohen’s Kappa (κ), indicating a high level of agreement (κ = 0.91).

### 2.5. Data Items

Two reviewers independently extracted study characteristics, population details, intervention or phenomenon, social outcomes examined, key findings, effect sizes, and relevant methodological notes. Discrepancies between reviewers were resolved through consensus.

### 2.6. Risk-of-Bias (Quality) Assessment

Tools were applied by two independent reviewers according to study design: for Qualitative Studies, the Joanna Briggs Institute (JBI) Critical Appraisal Checklist for Qualitative Research was used [23]; for Quantitative Observational Studies: The NIH Quality Assessment Tool for Observational Cohort and Cross-Sectional Studies were employed [24]; for Randomized Controlled Trials (RCTs), the Cochrane Risk of Bias tool (RoB 2.0) was used [25]; for Mixed-Methods Studies, the Mixed-Methods Appraisal Tool (MMAT) was used to appraise the relevant components [26]. Studies were not excluded based on quality assessment scores; instead, the assessment was used to inform the interpretation of the findings and discuss the strength of the evidence. A summary paragraph linking quality to confidence in findings is included in Section 3.3.

### 2.7. Thematic Analysis Procedures

Two reviewers (F.M., L.C.) independently performed line-by-line coding of all qualitative findings and relevant quantitative and mixed-methods summaries. The initial coding frameworks were subsequently compared to assess convergence, and discrepancies were minimal. Codes were then grouped into descriptive themes and further refined into analytical themes addressing the research question. Any disagreements were resolved through discussion; a senior reviewer (L.G.) was available but was not required to adjudicate disagreements. Rayyan was used to support the organization and traceability of codes [27].

### 2.8. Data Synthesis

Due to substantial heterogeneity across study designs, interventions, and outcome measures, a meta-analysis was not appropriate. A thematic synthesis was conducted to integrate findings across methodologies. This involved three iterative stages: first, a free line-by-line coding was performed to detail the meanings of outcomes and results. Then, based on the proposed codes, descriptive themes were extracted and were merged into single themes. Finally, from the descriptive themes, analytical ones were extracted to group all the themes. This method allows identification of mechanisms that transcend methodological differences [28].

## 3. Results

### 3.1. Study Selection

The systematic search and selection process are detailed in the PRISMA flow diagram (Figure 1). The initial database search yielded 416 records. After the removal of duplicates, the titles and abstracts of 146 records were screened. Of these, 60 were excluded for not meeting the eligibility criteria. The full texts of the remaining 26 articles were thoroughly assessed for eligibility. Five systematic reviews were excluded at this stage, as per the updated protocol. Furthermore, 5 additional studies identified from other sources were included, resulting in the final inclusion of 29 (27 reported empirical outcomes and 2 were protocols), primary studies that specifically addressed the interplay between physical activity and social barriers in PwMS. The high inter-rater reliability during full-text screening (κ = 0.91) between the two in-charged researchers underscores the consistency of the selection process.

### 3.2. Study Characteristics

Due to substantial heterogeneity across study designs, interventions, and outcome measures, a meta-analysis was not appropriate. A thematic synthesis was therefore conducted to integrate findings across methodologies, following three iterative stages: free line-by-line coding, development of descriptive themes, and generation of analytical themes. This approach allows the identification of mechanisms that transcend methodological differences. The characteristics of the 29 included studies are summarized in Table 2. The sample comprised 13 qualitative studies, 8 quantitative observational studies, 5 mixed-methods studies, and 3 randomized controlled trials (RCTs). Publication years ranged from 2004 to 2024, with studies conducted across Europe, North America, and Oceania. Sample sizes varied considerably, ranging from 9 to 146 participants. Interventions and phenomena also showed substantial variability and included group exercise programs (e.g., MOVE MS), aqua fitness, individually tailored exercise plans, adapted sports such as rock climbing, community-based yoga, home-based physical activity programs, and general explorations of physical activity experiences. Effect sizes were extracted or calculated when possible and are reported in Table 2. For studies lacking sufficient statistical information, this is explicitly noted in the table notes.

### 3.3. Risk-of-Bias Assessment

The detailed summary of the risk of bias evaluations are provided in Table 3, Table 4, Table 5 and Table 6 for qualitative, quantitative, RCT, and mixed-methods studies, respectively. Risk of bias was assessed only for empirical studies reporting outcomes; study protocols were therefore excluded from this assessment.

#### Interpretation of Risk-of-Bias Findings

Theme 1, concerning the multidimensional nature of social barriers, was supported predominantly by high-quality qualitative studies with a low risk of bias, including those by Adamson et al., Crank et al., Kayes et al., Russell et al., and Wolf et al. Moderate-quality quantitative studies also corroborated these findings. Overall, the convergence of evidence across study designs indicates a high level of confidence in this theme.

Theme 2, which conceptualizes physical activity as a mechanism for social inclusion, was supported mainly by low-risk qualitative studies and moderate-quality mixed-methods studies. The consistency of findings across different methodologies strengthens confidence in this theme, resulting in a moderate-to-high level of confidence in the robustness of the evidence.

Theme 3, addressing theoretical frameworks underpinning change, was supported by evidence from moderate-quality randomized controlled trials, quantitative correlational studies, and qualitative research. Some quantitative studies demonstrated a moderate risk of bias, primarily due to potential confounding factors. Consequently, while the findings related to this theme are considered reliable, they should not be interpreted as establishing causal relationships.

**Table 3:** Risk-of-Bias Assessment of Qualitative Studies (JBI)—Empirical Studies Only (*n* = 11).

Study	Philosophical Perspective	Methodology Congruence	Data Collection	Data Analysis	Researcher Reflexivity	Participant Voice	Ethical Approval	Conclusions Supported	Overall RoB
[30]	+	+	+	+	?	+	+	+	Low
[12]	+	+	+	+	?	+	+	+	Low
[6]	+	+	+	+	?	+	+	+	Low
[34]	?	+	+	+	?	+	+	+	Moderate
[36]	+	+	+	+	?	+	+	+	Low
[11]	?	+	+	+	?	+	+	+	Moderate
[20]	?	+	+	+	?	+	+	+	Moderate
[13]	+	+	+	+	?	+	+	+	Low
[16]	?	+	+	+	?	+	+	+	Moderate
[39]	+	+	+	+	?	+	+	+	Low
[46]	+	+	+	+	?	+	+	+	Low

Only qualitative studies reporting empirical outcomes were included. Study protocols were excluded from the risk of bias assessment. Legend (JBI): + = low risk; ? = unclear risk.

**Table 4:** Risk-of-Bias Assessment of Quantitative Observational Studies (NIH)—Empirical Studies Only (*n* = 7).

Study	Research Question	Population Defined	Participation Rate	Exposure Measured	Outcome Measured	Confounders	Statistical Analysis	Overall RoB
[31]	+	+	?	+	+	?	+	Low
[8]	+	+	?	+	+	+	+	Low
[9]	+	+	?	+	+	?	+	Moderate
[37]	+	+	+	+	+	+	+	Low
[40]	+	+	?	+	+	+	+	Low
[41]	+	+	?	+	+	+	+	Low
[14]	+	?	?	+	+	?	+	Moderate

Only quantitative observational studies reporting empirical outcomes were included. Study protocols and meta-analyses were excluded from this assessment. Legend (NIH): + = low risk of bias; ? = unclear risk of bias.

**Table 5:** Risk-of-Bias Assessment of Randomized Controlled Trials (RoB 2.0).

Study	Randomization	Deviations	Missing Data	Outcome Measurement	Selective Reporting	Overall RoB
[44]	?	+	+	+	+	Low

Only randomized controlled trials reporting outcome data were assessed. RCT protocols were excluded. Legend (RoB 2.0): + = low risk of bias; ? = some concerns.

**Table 6:** Risk-of-Bias Assessment of Mixed-Methods Studies (MMAT)—Empirical Studies Only (*n* = 4).

Study	Clear RQ	Rationale	Integration	Interpretation	Data Quality	Overall RoB
[35]	+	+	?	+	+	Moderate
[21]	+	?	?	+	+	Moderate
[42]	+	+	+	+	+	Low
[43]	+	+	?	+	+	Moderate

Only mixed-methods studies reporting empirical outcomes were included. Methodological papers were excluded from this assessment. Legend (MMAT): + = criterion satisfied; ? = criterion partially satisfied/unclear.

### 3.4. Thematic Synthesis

The thematic synthesis identified three analytical themes, each supported by multiple descriptive subthemes derived from the included studies. The themes reflect patterns consistently observed across qualitative, quantitative, and mixed-methods evidence [12,13,30,37].

#### 3.4.1. Theme 1: The Multidimensional Nature of Social Barriers to PA

This theme describes the variety of social, interpersonal, and environmental barriers that limit PA participation among PwMS.

Isolation and Lack of Support

Many participants reported insufficient guidance from healthcare professionals or a lack of social support from peers and family members, contributing to uncertainty and reduced confidence in engaging in PA [12,13]. Several studies also highlighted an interaction between symptom burden (e.g., pain and fatigue) and social withdrawal, whereby reduced social participation further limited engagement in PA [20,37,40,47].

Structural and Environmental Barriers

Inaccessible facilities, unsuitable environments, transportation challenges, and financial constraints were frequently cited as obstacles to participation across studies [13,14,16,30,31,34,45,48].

Stigma and Identity-Related Barriers

Several studies described experiences of perceived social exclusion and stigma, as well as internalized beliefs aligned with a “sick role,” which discouraged engagement in PA [12,13].

These findings represent descriptive outputs from the included studies and do not include interpretation beyond direct thematic coding.

#### 3.4.2. Theme 2: Physical Activity as a Mechanism for Social Inclusion

Across study designs, PA, particularly in group-based contexts, was associated with reductions in social barriers and improvements in social experiences [30,37,40].

Peer Support and Shared Experiences

Group settings frequently fostered positive social interactions, shared understanding, and a sense of universality. Participants valued the opportunity to connect with others who shared similar challenges, reporting reduced feelings of isolation and increased acceptance within PA contexts [29,30,39,46].

Role of Knowledgeable Professionals

Trainers and instructors with MS-specific knowledge were perceived as supportive, trustworthy, and essential to creating an emotionally safe environment for participation, particularly by providing reassurance and appropriate exercise adaptations [12,30,38,44].

Enhanced Social Connectedness

Participation in PA was associated with increased social interaction, acceptance, and a sense of belonging within group contexts [21,30,37].

#### 3.4.3. Theme 3: The Role of Theoretical Frameworks in Explaining Social Change

Several studies explicitly or implicitly supported theoretical frameworks such as Social Cognitive Theory (SCT). Specifically, social support and professional guidance were shown to influence self-efficacy [12,44], while higher levels of self-efficacy were associated with greater physical activity participation and reduced perceived barriers [40]. In addition, environmental and personal factors were found to interact dynamically to shape behavior across different contexts [35,37,40,42,49,50].

#### 3.4.4. Conceptual Model

**Figure 2:**
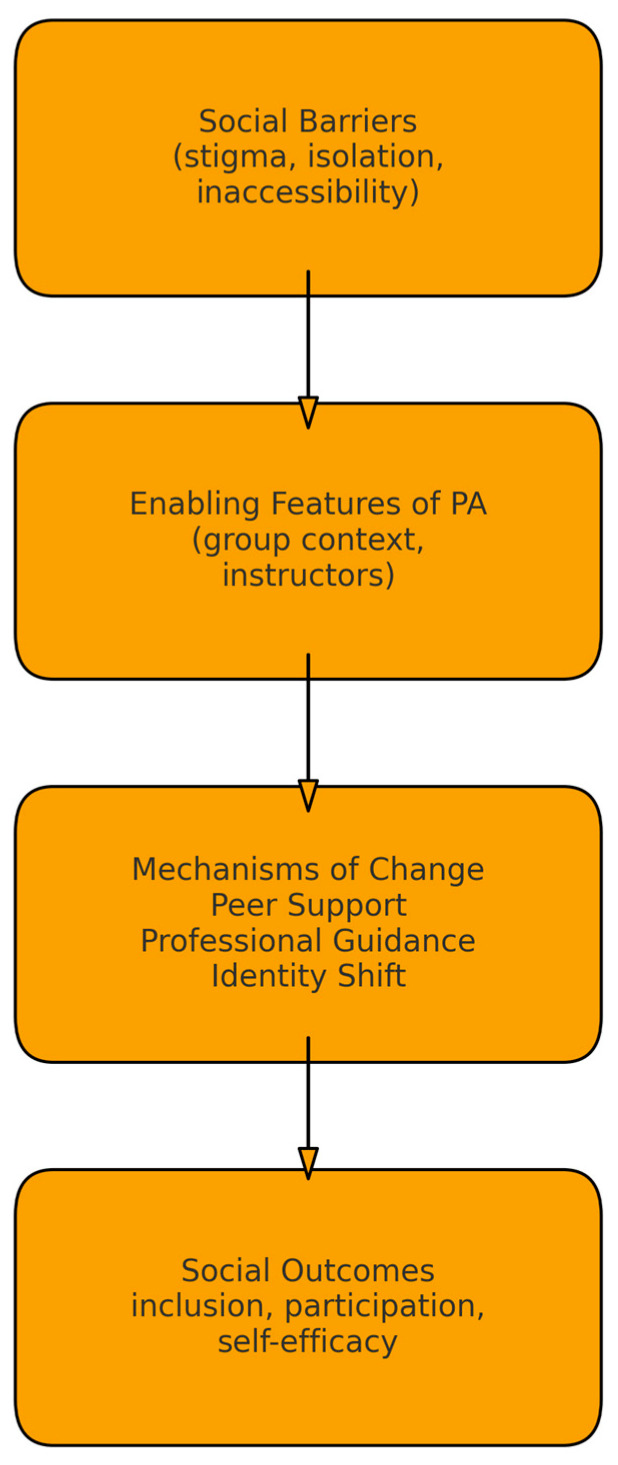
Conceptual Model of Thematic Synthesis Conceptual model illustrating the mechanisms through which physical activity reduces social barriers in people with multiple sclerosis.

Figure 2 presents a conceptual model that integrates the identified social barriers, the enabling features of physical activity interventions, the mechanisms influencing change, and the resulting social outcomes. The model visually synthesizes the relationships identified during thematic analysis [30,37].

## 4. Discussion

This systematic review synthesized evidence from 29 primary studies to elucidate how physical activity (PA) and sport function as mechanisms through which people with multiple sclerosis (PwMS) overcome social barriers [12,13,30,37,51]. Unlike previous reviews that primarily enumerated types of barriers or facilitators, this review clarifies the mechanisms of change by which PA reinforces social inclusion, integrating findings across qualitative, quantitative, and mixed-methods studies [30,37]. The evidence demonstrates that PA, particularly when delivered in group-based, structured, and professionally supervised formats, operates far beyond a physical or biomedical intervention. It serves as a meaningful social and psychological catalyst, enabling PwMS to counteract isolation, challenge stigma, build supportive relationships, and experience shifts in identity that encourage deeper and more sustained engagement in community life [12,30,37].

### 4.1. Peer Support as a Central Social Mechanism

Across multiple high-quality qualitative studies with low risk of bias, group-based PA interventions consistently facilitated the development of peer support networks. Participants reported experiencing a sense of “universality,” mutual understanding, and shared challenge [29,30,39]. These interactions normalized MS-related symptoms, reduced feelings of isolation, and fostered a supportive social environment [12,13,37]. The importance of peer support was corroborated by moderate-quality quantitative studies, where social support was positively associated with higher PA levels and stronger self-efficacy [37,40,41]. Although causal interpretations are limited, several studies reported medium effect sizes for associations between social support and PA engagement, indicating that peer dynamics may meaningfully influence participation patterns [40,41].

### 4.2. The Role of Knowledgeable Professionals

A consistent and robust theme across study designs was the significance of instructors and health professionals skilled in MS-specific exercise prescription [12,13,30]. Their role extended beyond technical supervision to include: creating a psychologically safe environment [12,30] offering credible reassurance [12,44] adapting activities to fluctuating symptoms [30] challenging overprotectiveness or internalized stigma [13,30]. Participants frequently cited these supportive relationships as pivotal in initiating or sustaining PA participation [12,30]. This finding is supported by high-quality qualitative evidence and moderate-quality intervention studies [30,44]. Effect size reporting from quantitative studies was limited, but available data suggest small-to-moderate impacts of instructor-led interventions on social outcomes, reflecting the complexity of measuring social constructs in controlled designs [40,41].

### 4.3. Identity Transformation and Psychological Change

One of the most profound mechanisms identified was the shift in social identity that emerged through PA participation [36,39,43]. Engagement in structured programs allowed individuals to move away from a passive “patient identity” toward identities such as:

“exerciser” [36,43]

“athlete” [39]

“capable person” [36,37]

These identity shifts align with theoretical constructs from Social Cognitive Theory (SCT), particularly self-efficacy and observational learning within social environments [17]. Qualitative studies described this transformation as foundational for increasing confidence and motivation [36,39], while longitudinal quantitative findings indicated that higher self-efficacy was associated with reductions in perceived barriers, with small-to-moderate effect sizes [37,40,41]. Importantly, identity change also enhanced social participation beyond PA contexts, suggesting broad psychosocial benefits of exercise engagement [36,37,43].

### 4.4. Theoretical Integration: Social Cognitive and Socio-Ecological Perspectives

The observed mechanisms of change map coherently onto SCT’s triadic model, wherein personal factors, environmental conditions, and behaviors interact dynamically [17]. Social support and professional guidance function as environmental determinants that increase self-efficacy, which in turn predicts PA participation [40,41]. Complementarily, socio-ecological frameworks emphasize that participation is strongly shaped by structural and environmental barriers [18]. This review confirms that even individuals with strong motivation and adequate knowledge may remain excluded when facilities, transportation, or financial resources are insufficient [13,14,30]. Together, these models provide a comprehensive explanation for why PA can meaningfully counteract social barriers only when socially oriented elements, group dynamics, trained instructors, accessible environments, are deliberately integrated into program design [18,30,37].

### 4.5. Cultural, Structural, and Severity-Related Considerations

Most included studies were conducted in Western, high-income countries, where community-based exercise programs and adapted PA infrastructures are more accessible [14,30]. This geographic concentration may limit generalizability, as cultural attitudes toward disability vary [18] access to adapted PA programs differs substantially across regions [14,30] stigma may be more pronounced in certain cultural contexts [12,13]. Furthermore, the majority of studies involved participants with mild-to-moderate disability (EDSS < 6.5) [37,40]. Social and environmental barriers for individuals with more severe disability were likely underrepresented, and their experiences may differ significantly, for example, requiring personal support workers, specialized equipment, or adapted transportation [13,14,30]. Finally, while qualitative evidence offers rich insights, the relative scarcity of high-quality RCTs and implementation studies limits the ability to infer causality or long-term sustainability of benefits [40,41].

### 4.6. Integration of Quantitative Findings and Effect Sizes

Where quantitative data were available, effect sizes related to social outcomes (self-efficacy, social participation, stigma reduction) were generally small-to-moderate [40,41]. Although effect sizes were often not reported in primary studies, and occasionally could not be calculated due to incomplete data, those that were available corroborated qualitative findings [37,40]. Statistically significant improvements were most commonly observed in self-efficacy [40,41], perceived social support [30,37], and social participation [36,37]. However, statistical non-significance did not necessarily indicate absence of effect; rather, some studies were underpowered or used heterogeneous measures [40]. Overall, although effect sizes were moderate, their consistency across methodologies strengthens confidence in the robustness of the identified mechanisms [30,37,41]. This discussion integrates methodological insights, thematic findings, and quantitative evidence to illustrate how PA can mitigate social barriers for PwMS. The mechanisms are interdependent, peer support, professional guidance, and identity transformation operate simultaneously and dynamically, reinforcing one another to create meaningful social change [17,30,37].

## 5. Limitations

Several limitations of this review and of the underlying evidence base must be considered when interpreting the findings. First, the predominance of qualitative studies, while offering rich insights into lived experiences, limits the ability to quantify the magnitude of effects or establish causal pathways between physical activity (PA) and social outcomes. Quantitative studies that did assess social variables frequently employed heterogeneous measurement tools, making comparison difficult and preventing meta-analytic synthesis.

Second, the majority of included studies were conducted in Western, high-income countries, where access to adapted PA programs, trained professionals, and community resources may be more readily available. This geographic and cultural concentration limits generalizability to regions where stigma may be more pervasive, community infrastructures less developed, or social norms differ substantially.

Third, most studies sampled individuals with mild-to-moderate disability (EDSS < 6.5). People with severe mobility limitations or progressive forms of MS were largely underrepresented, likely leading to an incomplete picture of structural, environmental, and social barriers relevant to more disabled populations [52].

Finally, few studies employed longitudinal or implementation-focused designs. As a result, evidence regarding long-term sustainability of social benefits, real-world feasibility of PA programs, and scalability within clinical or community contexts remains limited.

These limitations highlight the need for caution in interpreting certain findings while also pointing to clear directions for future research.

## 6. Implications for Practice and Research

### 6.1. Clinical and Practical Implications

Findings from this review suggest several actionable recommendations for clinicians, exercise professionals, and program developers. Rather than merely advising people with multiple sclerosis (PwMS) to be physically active, clinicians should prioritize socially oriented exercise prescriptions through social prescribing approaches, directing individuals to structured, group-based physical activity programs where social support and identity-building mechanisms can develop. In parallel, investment in specialized professional training is essential, as instructors and healthcare professionals should be equipped with competencies in MS-specific exercise prescription, symptom fluctuation management, group facilitation, and communication skills aimed at fostering psychological safety and empowerment.

Program design should intentionally maximize interaction and inclusion by incorporating peer-to-peer engagement, shared goals, and opportunities for mutual support. Practical considerations, including facility accessibility, pool entry methods, changing room layout, and transport availability, must be addressed to ensure genuine inclusion. Finally, advocacy for structural and policy support is crucial, as transportation services, funding for personal support workers, and subsidies for adapted physical activity programs may be essential to enable participation for individuals with greater disability or financial constraints.

### 6.2. Research Implications

Findings from this review suggest several actionable recommendations for clinicians, exercise professionals, and program developers. Rather than merely advising people with multiple sclerosis to “be active,” clinicians should prioritize socially oriented exercise prescriptions through social prescribing approaches, directing individuals to structured, group-based physical activity programs where social support and identity-building mechanisms can develop. In parallel, investment in specialized professional training is essential. Instructors and healthcare professionals should receive education in MS-specific exercise prescription, symptom fluctuation management, group facilitation, and communication skills aimed at fostering psychological safety and empowerment.

## 7. Conclusions

This systematic review demonstrates that physical activity, when thoughtfully designed and delivered, functions as far more than a physiological intervention for people with multiple sclerosis. Across a diverse body of qualitative, quantitative, and mixed-methods evidence, PA emerges as a social catalyst capable of rebuilding connections, enhancing social identity, and reducing barriers rooted in stigma, exclusion, or environmental limitations. The most effective interventions consistently included group-based formats; knowledgeable and empathetic professionals; opportunities for shared experience; and environments that support inclusion and capability. These elements work together to create conditions in which PwMS can experience meaningful social participation, greater confidence, and improved quality of life. By prioritizing group dynamics, professional support, and accessible environments, future PA programs can unlock the full social potential of physical activity and contribute to a more inclusive and empowering landscape for individuals living with MS.

## Figures and Tables

**Table 1:** Inclusion and exclusion criteria.

Criteria	Inclusion	Exclusion
**Population**	Adults (≥18) with clinically confirmed MS (PwMS)	Studies on other neurological populations or mixed populations without separate analysis for PwMS
**Intervention/Phenomenon**	Any PA, exercise, sport; social barriers/facilitators	Studies without a social component; solely pharmacological interventions
**Comparator/Context**	Any comparator or context	No exclusion based on comparator
**Outcomes**	Social outcomes (support, inclusion, stigma, identity)	Solely clinical, biological, or non-social psychological outcomes
**Study Designs**	Qualitative, quantitative, mixed-methods, RCTs	Systematic reviews, editorials, opinion pieces, non-peer-reviewed literature, non-English publications

**Table 2:** Characteristics of Included Studies (*n* = 29).

Study	Study Design	Population	Intervention/Phenomenon	Key Social Findings	Effect Size (ES)
[29]	Feasibility	PwMS (*n* = 33)	MOVE MS group program	Strong peer interaction; improved social identity	—(qualitative)
[30]	Qualitative	PwMS	Aqua fitness (AF)	Universality; reduced stigma; environmental barriers	—
[31]	Quantitative	Adults w/disability	Social participation factors	Socioenvironmental predictors strongest	*r* = 0.32 (reported)
[32]	RCT Protocol	PwMS	Step It Up (SCT-based)	Baseline data: social factors relevant	—(protocol)
[12]	Qualitative	PwMS (*n* = 19)	Tailored exercise	Peer support + knowledgeable HCPs	—
[33]	Protocol	Young PwMS	ESPRIMO intervention	Baseline context on stigma	—
[6]	Qualitative	PwMS	HRQoL facilitators	Social support essential	—
[34]	Qualitative	PwMS	PA barriers	Social + psychological barriers	—
[35]	Mixed-Methods	PwMS (*n* = 67)	Motivation + PA	Social support predicts PA	*r* = 0.41 (reported)
[8]	Cross-sectional	PwMS (*n* = 146)	SCT variables	Support correlates with light PA	*β* = 0.27; *p* < 0.05
[36]	Qualitative	PwMS (*n* = 15)	Adapted rock climbing	Enhanced social identity + belonging	—
[11]	Qualitative	PwMS (*n* = 21)	Barriers/facilitators	Environmental inaccessibility key	—
[10]	Meta-analysis	PwMS	PA levels vs. controls	PwMS less active globally	Included for contextual quantitative comparison, not as primary evidence
[9]	Quantitative	PwMS	Objective PA	PA influenced by social context	—(insufficient data)
[37]	Longitudinal	PwMS	Social integration	Higher social integration = better health	*β* = 0.34
[38]	Trial	PwMS (*n* = 18)	Blue Prescription	Combined support reduces barriers	*d* = 0.36 (calculated)
[20]	Qualitative	PwMS (*n* = 10)	PA experiences	Social influences on PA	—
[21]	Mixed-Methods	PwMS	Social participation	Multiple social dimensions explored	—
[13]	Qualitative	PwMS and HCPs (*n* = 32)	HCP roles	HCP support crucial for adherence	—
[16]	Qualitative	Disabilities	Environmental barriers	Attitudinal obstacles significant	—
[39]	Qualitative	PwMS (*n* = 19)	SCT-based PA	Positive identity shifts	—
[40]	Observational	PwMS	Barriers (BHADP)	Self-efficacy reduces barriers	*r* = −0.29
[41]	Secondary analysis	PwMS (*n* = 48)	SCT + PA	SCT explains heterogeneity	—(insufficient for ES)
[42]	Mixed-Methods	Wheelchair PwMS (*n* = 26)	SCT-guided	Social support essential	—
[43]	Mixed-Methods	PwMS (*n* = 25)	Community yoga	Improved social connectedness	—
[44]	RCT	PwMS (*n* = 30)	Wii home-based	Social well-being improved	*η^2^* = 0.21
[14]	Cross-sectional	Moderate-severe MS (*n* = 48)	Barriers to PA	Social + environmental barriers	—
[45]	Methodological	Disabilities	Barrier measure	Developed social barrier tool	—
[46]	Qualitative	PwMS (*n* = 10)	Multimodal exercise	Strong group support	—

Note. (i) Two records were study protocols and did not report empirical outcomes. (ii) One meta-analysis was retained for contextual quantitative comparison only and did not contribute to the thematic synthesis or risk-of-bias assessment. Abbreviations: PwMS: People with Multiple Sclerosis; PA: Physical Activity; HCPs: Healthcare Professionals; SCT: Social Cognitive Theory; AF: Aqua fitness; HRQoL: Health-Related Quality of Life; RCT: Randomized Controlled Trial; BHADP: Barriers to Health Activities among Disabled Persons scale; MS: Multiple Sclerosis.

## Data Availability

No new data were created or analyzed in this study. Data sharing is not applicable to this article.

## Supplementary Materials

The following supporting information can be downloaded at: https://www.mdpi.com/article/10.3390/sports14010025/s1, Table S1. Search Strategy; supplementary-checklist.

## Abbreviations

The following abbreviations are used in this manuscript:

Abbreviation	Meaning
MS	Multiple Sclerosis
PwMS	People with Multiple Sclerosis
PA	Physical Activity
SCT	Social Cognitive Theory
PWB	Psychological Well-Being
HCPs	Healthcare Professionals
HRQoL	Health-Related Quality of Life
RCT	Randomized Controlled Trial
AF	Aquafitness
