# Supplementary material for: Physical Activity as a Tool for Social Inclusion in Multiple Sclerosis: A Systematic Review of Qualitative, Quantitative, and Mixed-Methods Evidence
Source: Sports (Basel). 2026 Jan 5;14(1):25. doi: 10.3390/sports14010025 (PMC12845790; doi:10.3390/sports14010025)
Supplement: Supplementary file 1 [file sports-14-00025-s001.zip › sports-4037056-supplementary/sports-4037056 - Supplementary S1.docx]

## ****Supplementary Table S1. Search Strategy****

| **Research Question** | **Databases** | **Search String** |
| --- | --- | --- |
| **How does physical activity or exercise help people with multiple sclerosis overcome social barriers?** | **PubMed** | (“multiple sclerosis”[Title/Abstract]) AND (“physical activity”[Title/Abstract] OR exercise[Title/Abstract]) AND (“social barriers”[Title/Abstract]) |
|  | **Scopus** | TITLE-ABS-KEY(“multiple sclerosis” AND (“physical activity” OR exercise) AND “social barriers”) |
|  | **Web of Science** | TS=(“multiple sclerosis”) AND TS=(“physical activity” OR exercise) AND TS=(“social barriers”) |

- The search strategy was based on four core concepts: multiple sclerosis, physical activity, exercise, and social barriers.
- Search strings were adapted to the syntax of each database.
- Limits applied: English language, peer-reviewed articles, adult population.
