# Supplementary material for: Physical Activity as a Tool for Social Inclusion in Multiple Sclerosis: A Systematic Review of Qualitative, Quantitative, and Mixed-Methods Evidence
Source: Sports (Basel). 2026 Jan 5;14(1):25. doi: 10.3390/sports14010025 (PMC12845790; doi:10.3390/sports14010025)
Supplement: Supplementary file 1 [file sports-14-00025-s001.zip › sports-4037056-supplementary/sports-4037056-supplementary-checklist.pdf]

Physical activity as a tool for social inclusion in multiple sclerosis: A Systematic Review

Section / Topic	Ite	PRISMA 2020 item	How it is reported in the manuscript
**TITLE**	1	Identify the report as a systematic review.	Title: *"Physical activity as a tool for social inclusion in multiple sclerosis: A Systematic Review"* explicitly labels the study as a systematic review.
**ABSTRACT**	2	Structured summary following PRISMA for Abstracts.	Structured abstract reporting background, objectives, methods (databases, time frame, eligibility), results (number of studies; main themes), and conclusions (implications for social inclusion in PwMS). *(To be checked against journal abstract structure; can be aligned with PRISMA for Abstracts if needed.)*
**INTRODUCTION – Rationale**	3	Describe the rationale for the review in the context of existing knowledge.	Introduction explains the need to understand how PA overcomes social barriers in PwMS and highlights the gap that previous reviews did not specifically address PA as a mechanism for social inclusion.
**INTRODUCTION – Objectives**	4	Provide an explicit statement of the objectives or questions.	The research question is explicitly formulated: *"How does physical activity or exercise help people with multiple sclerosis overcome social barriers?"*
**METHODS – Eligibility criteria**	5	Specify inclusion and exclusion criteria and how studies were	Eligibility criteria defined using a PICOS framework (Population, Intervention, Comparator, Outcomes, Study Design), with explicit exclusion criteria (language, non-original data, non-social outcomes). Reported in Section 2.2 and Table 1.
**METHODS – Information sources**	6	Specify all information sources and date of last search.	Section 2.3: databases (PubMed, Scopus, Web of Science) and use of Zotero are described; last search on 31 October 2025; time frame from January 1997 onwards.
**METHODS – Search strategy**	7	Present full search strategies for all databases.	Section 2.3 summarises search concepts (MS, PA/exercise, social barriers), Boolean operators and search fields ("title and abstract", "TITLE-ABS-KEY", "topic"). Full detailed strings could be added as Supplementary Material if requested.
**METHODS – Selection process**	8	Specify the methods used to select studies.	Section 2.4: selection conducted in Rayyan by two independent reviewers, with duplicate removal, two-step screening (title/abstract, full text), consensus procedures, third reviewer, and Cohen's $\kappa = 0.91$. PRISMA flow diagram referenced as Figure 1.
**METHODS – Data collection process**	9	Specify methods used to collect data from reports.	Section 2.5: data extracted independently by two reviewers using a standardized Excel form; discrepancies resolved by cross-checking and discussion.
**METHODS – Data items (outcomes)**	10a	List and define all outcomes for which data were sought.	Section 2.2 and 2.5: primary outcomes defined as social outcomes (social support, social inclusion/exclusion, stigma, social identity, community participation).
**METHODS – Data items (other variables)**	10b	List and define all other variables and how missing data were	Section 2.5 and Table 2: additional variables include study design, country, sample characteristics, type of PA intervention/phenomenon, key social findings. Handling of missing data is not extensively described; interpretation is informed by available data.

**METHODS – Study risk of bias assessment**	11	Specify methods to assess risk of bias.	Section 2.6: risk of bias assessed with design-specific tools (JBI, NIH tool, RoB 2.0, MMAT); two independent reviewers; results used to inform interpretation rather than exclude studies.
**METHODS – Effect measures**	12	Specify effect measures for each outcome.	Section 2.7: meta-analysis deemed inappropriate due to heterogeneity; thematic synthesis used instead. No pooled quantitative effect measures are reported.
**METHODS – Synthesis methods**	13	Describe methods to synthesise results.	Section 2.7: thematic synthesis with three steps (line-by-line coding, descriptive themes, analytical themes) following Thomas & Harden; Rayyan used to support coding.
**METHODS – Reporting bias assessment**	14	Describe methods to assess risk of bias due to missing results.	No formal statistical assessment (e.g., funnel plot) is described; potential bias is considered qualitatively in the Discussion when interpreting the strength and variability of evidence.
**METHODS – Certainty assessment**	15	Describe methods to assess certainty of evidence.	No formal GRADE (or similar) procedure is reported. Certainty/strength of evidence is discussed narratively in the Discussion, with reference to study quality and heterogeneity.
**RESULTS – Study selection**	16a	Describe results of the search and selection, ideally with flow	Section 3.1 and Figure 1: initial records (n = 416), records after duplicate removal (n = 77), records excluded at screening (n = 60), full-texts assessed (n = 26), systematic reviews excluded (n = 5), additional sources (n = 5), and final studies included (n = 31).
	16b	Cite studies that appeared to meet criteria but were excluded, and	Section 3.1: systematic reviews (n = 5) are identified as excluded at full-text because they did not match the updated protocol focusing on primary studies. Specific references can be listed in Supplementary Material if required.
**RESULTS – Study characteristics**	17	Present characteristics of each included study.	Section 3.2 and Table 2: study design, country, population, intervention/phenomenon, and key social findings for all 31 studies.
**RESULTS – Risk of bias in studies**	18	Present assessments of risk of bias for each included study.	Section 3.3 and Table 3 (sub-tables 3.1–3.4) report risk of bias judgments for qualitative, quantitative, RCT, and mixed-methods studies, using design-appropriate tools.
**RESULTS – Results of individual studies**	19	For all outcomes, present summary statistics and effect estimates.	Individual study results are summarized narratively and in Table 2 through key social findings (e.g., social support, identity change, environmental barriers). No pooled effect sizes are reported due to methodological heterogeneity.
**RESULTS – Results of syntheses**	20	Present results of all statistical or qualitative syntheses.	Section 3.4: thematic synthesis results with three analytical themes describing social barriers, mechanisms by which PA supports inclusion, and identity/social role changes. The conceptual model is presented in Figure 2.
**RESULTS – Reporting bias**	21	Present assessment of risk of bias due to missing results.	Potential influence of publication and reporting biases is discussed within the limitations of the evidence base in the Discussion (e.g., variability of designs, incomplete control for confounding, Western-centric samples).

**RESULTS – Certainty of evidence**	22	Present assessment of certainty for each outcome.	Certainty is not quantified with GRADE; instead, the Discussion comments on the strength and consistency of evidence across themes and study designs.
**DISCUSSION – General interpretation**	23a	Provide a general interpretation of results in context of other	Discussion interprets how PA can function as a socially transformative mechanism for PwMS and situates findings within existing literature on PA, social barriers, and disability models.
**DISCUSSION – Limitations of evidence**	23b	Discuss limitations of the included evidence.	The Discussion notes limitations such as heterogeneity of designs and interventions, variability in risk of bias, and partial coverage of MS phenotypes and disability levels.
**DISCUSSION – Limitations of review process**	23c	Discuss limitations of the review methods.	The review acknowledges constraints such as restriction to English-language studies and methodological heterogeneity that prevented meta-analysis.
**DISCUSSION – Implications**	23d	Discuss implications for practice, policy, and future research.	The Discussion outlines implications for designing PA interventions that are socially oriented, and suggests directions for future work on stigma, identity, and structural barriers in PwMS.
**OTHER – Registration and protocol**	24a–c	Provide registration details, protocol access, and amendments.	The review followed a structured protocol but was **not registered** in a public database; this is clarified in the checklist and should be explicitly stated in the Methods (e.g., “The review protocol was not prospectively registered.”).
**OTHER – Support**	25	Describe sources of support and role of funders.	Funding statement included in the manuscript (Funding section).
**OTHER – Competing**	26	Declare competing interests.	Conflicts of Interest section: authors declare no conflicts of interest.
**OTHER – Availability of data, code, and**	27	Report where data, code, and other materials are available.	Data availability is not explicitly described as a separate statement. For the checklist you can note: *“Not explicitly reported in the manuscript; data extraction sheets and synthesized data can be made available from the corresponding author upon reasonable request.”* (da aggiungere nella versione rivista, se concordi).
